# Sociodemographic and psychological characteristics of school shooters in the United States: a systematic review of the literature

**DOI:** 10.3389/fpsyt.2025.1735929

**Published:** 2026-01-12

**Authors:** Michela Minelli, Angelo Zappalà, Silvia Vayr, Pekka Santtila

**Affiliations:** 1 Istituto Universitario Salesiano di Torino (IUSTO) - Salesian University Institute Torino Rebaudengo, Torino, Italy; 2 Åbo Akademi University, Turku, Finland; 3 CBT ACADEMY, Torino, Italy; 4 Faculty of Arts and Sciences, New York University (NYU) Shanghai, Shanghai, China

**Keywords:** gun violence, homicide, mass shooting, school shooters, school shootings, school violence

## Abstract

**Background:**

School shootings represent a complex and critical phenomenon observed worldwide, though their frequency is particularly high in the United States compared to other developed countries. A systematic examination of the socio-demographic and psychological characteristics of school shooters is essential for identifying potential recurring patterns and advancing scholarly understanding of the phenomenon. Nonetheless, the current body of evidence remains fragmented and inconsistent.

**Methods:**

A systematic literature search was conducted across PubMed, PsycINFO, and Web of Science databases covering publications from 1900 to 2024. From an initial pool of 1,862 publications, 13 studies meeting inclusion criteria were selected for analysis. The review specifically focused on socio-demographic, psychological, and behavioral characteristics of school shooters, including age, gender, family background, social experiences, mental health, and risk behaviors.

**Results:**

The findings indicated that approximately 75% of the characteristics we aimed to analyze were not available in the reviewed literature. Despite these data limitations, some recurrent features emerged, such as social isolation, traumatic experiences (e.g., bullying, dysfunctional families), and problematic psychological traits (e.g., depression, narcissism, lack of empathy). Rather than revealing a single perpetrator typology, the evidence suggests the existence of multiple heterogeneous patterns shaped by individual differences and diverse contributing factors.

**Discussion:**

The scarcity and fragmentation of data are linked to methodological heterogeneity and the lack of standardized protocols for data collection and analysis. Future research should prioritize the development of uniform methodologies to improve data quality and comparability. This would facilitate a deeper understanding of the phenomenon and strengthen preventive strategies. The review underscores the urgency of overcoming current research dispersion to identify meaningful patterns and dynamics.

## 1 Introduction

School shootings represent a complex and multidimensional phenomenon that has profoundly shaped the social and educational landscape of the United States (1). Although similar incidents have occurred in other countries (2–11), their prevalence in the United States is markedly higher, constituting a distinct form of “American exceptionalism” (12). A CNN analysis revealed that between 2009 and 2018, the U.S. experienced 288 school shootings, compared to only five in the other G7 countries combined (Canada, France, Germany, Japan, Italy, and the United Kingdom) (13). According to Rowhani-Rahbar and Moe (12), the U.S. has witnessed 57 times as many school shootings as all other major industrialized nations combined.

The literature nonetheless converges on the absence of a single perpetrator profile: shooters vary widely in age, ethnicity, gender, academic achievement, and social characteristics (14–17; O’Toole, 2000). However, several recurrent risk factors have been identified, such as adverse childhood experiences, emotional distress, bullying, exposure to violence, and firearm availability (18–20). Given the low incidence of such events, criminological methods face inherent challenges, and school shootings are often studied within the broader framework of interpersonal violence (21).

To sum, empirical research investigating the characteristics of school shooters has consistently identified clusters of sociodemographic features, psychological traits, and life experiences that appear with notable frequency in school shooter profiles. While acknowledging the individual uniqueness of each case, these studies reveal convergent patterns that can be systematically organized into three interconnected domains: sociodemographic characteristics, psychological and mental health characteristics, and precipitating life experiences and life events.

### 1.1 Sociodemographic characteristics

Although each school shooter’s life story is distinct, scholars have identified recurring sociodemographic patterns among perpetrators. Research suggests that most school shooters are white men from middle to lower-middle-class backgrounds (22–24), often described as socially isolated “loners” (25–29). Many also experience academic difficulties or failure prior to their attacks (30).

### 1.2 Psychological traits and mental health characteristics

School shooters frequently exhibit distinct psychological profiles and mental health symptoms. Many display symptoms of depression (31–33) and often show problematic personality traits, including unstable self-esteem (27), narcissistic tendencies (27, 28, 34, 35), poor coping or anger management skills (26, 28), and paranoid thinking patterns (36). One study found that many school shooters demonstrated an obsession with violence (29), while two studies reported suicidal ideation among perpetrators (32).

### 1.3 Precipitating experiences and life events

Numerous adverse life experiences and traumatic events are frequently reported in the backgrounds of school shooters. These include experiences of social rejection and ostracism (26), bullying victimization (26, 28, 29, 33, 35, 37, 38), and family dysfunction or abuse (26–29, 32). Many school shooters also experience significant personal losses or failures (29, 39) and often undergo a major triggering event before carrying out an attack (26–29, 33, 38).

Despite these converging findings from individual studies across multiple domains, the existing literature remains fragmented, with researchers employing different methodologies, definitions, and analytical frameworks. Moreover, a systematic and comparative collection of school shooters’ characteristics remains lacking.

To address this gap, we conducted a systematic review guided by the PICOS framework. Our research question was: Among individuals who perpetrated school shootings in the United States, what sociodemographic, psychological, behavioral, and life-experience characteristics are reported in primary peer-reviewed empirical studies? No intervention or comparator group was specified, as the focus of this review was descriptive rather than comparative or evaluative.

## 2 Methods

### 2.1 Literature sources

The research was conducted using electronic databases accessed through the EBSCOhost platform. The specific databases consulted included: Atlas Religion Database with AtlaSerials, MLA Directory of Periodicals, Library, Information Science & Technology Abstracts, Communication & Mass Media Complete, GreenFILE, Regional Business News, European Views of the Americas: 1493 to 1750, Art Full Text (H.W. Wilson), eBook Collection (EBSCOhost), Energy & Power Source, MEDLINE, MLA International Bibliography with Full Text, ERIC, Teacher Reference Center, APA PsycInfo, APA PsycArticles, Philosopher’s Index with Full Text, CINAHL with Full Text, Academic Search Complete, Business Source Ultimate, Historical Abstracts with Full Text, OpenDissertations, RILM Abstracts of Music Literature, Education Research Complete, and the eBook Open Access (OA) Collection (EBSCOhost).

### 2.2 Search string and filters

The following search terms were selected and applied to the databases. Several trials and iterations were conducted, testing different keywords and Boolean operator combinations, in order to determine the most effective and efficient strategy. The goal was to identify a search string broad enough to yield a sufficiently large number of results, while still ensuring coherence and manageability. For this reason, all search terms were in English.

The final search string was:

“school shooter” OR “school shooters” OR “school shooting” OR “school shootings” OR “school rampages” OR “school rampage” OR “active shooter” OR “active shooters” OR “public school shooting” OR “public school shootings” OR “public school shooter” OR “public school shooters”.

This string was implemented to search within article titles OR abstracts. This choice was made to maximize the likelihood of retrieving relevant publications. Additionally, several filters were applied to refine the results. Specifically, the search was limited to academic journal publications subjected to peer review, ensuring a focus on high-quality and reliable studies. The temporal scope was set between 1900 and 2024. However, results were only retrieved from 1979 onward, most likely due to limitations in historical database coverage. As of September 23, 2024, the total number of articles retrieved was *n* = 1862.

### 2.3 Screening

Following PRISMA guidelines, the identification process began with the import of all records. A first step involved the removal of 567 duplicates or supplementary records, leaving 1295 articles eligible for abstract screening.

Abstracts were independently reviewed by three reviewers, one of whom also served as the lead reviewer coordinating the process. This triple-blind evaluation resulted in the exclusion of 1228 articles, reducing the pool considerably. Consequently, 67 articles were identified for full-text assessment. Full texts could not be retrieved for 8 articles despite attempts, leaving 59 available for review. Of these, 45 were excluded after detailed examination, resulting in 14 studies meeting the inclusion criteria. Ultimately, one article was further excluded due to missing methodological details (e.g., sample construction), as these were referenced in an unpublished supplementary document.

Disagreements among reviewers were resolved through discussion until unanimity was achieved. Overall, 73% of the articles (*n* = 942) were classified without conflict, while 27% (*n* = 353) initially presented conflicts that were subsequently resolved through consensus. No unresolved conflicts remained, as agreement was always reached. It should be emphasized that this measure does not constitute a formal reliability index (e.g., Cohen’s kappa) but rather provides an indicative overview of agreement.

The systematic selection process resulted in the exclusion of a substantial number of articles that did not meet the established inclusion criteria. Exclusions were primarily due to six main reasons: first, secondary studies such as reviews, meta-analyses, and commentaries were excluded in favor of primary empirical research; second, we excluded studies addressing school shootings outside the US, as well as those based on datasets that merged US and non-US cases; third, investigations with sample sizes smaller than ten shooters were deemed insufficient for meaningful analysis; fourth, research addressing school violence that did not involve firearms was excluded to maintain focus on shooting incidents specifically; fifth, only peer-reviewed academic journal articles were included, excluding grey literature, dissertations, and non-academic publications; and sixth, studies that failed to provide extractable socio-demographic and psychological characteristics of perpetrators were excluded as they did not contribute data relevant to the review objectives.

As previously noted, the 8 articles for which full texts were not retrievable (listed separately at the end of the references) were excluded, not because they failed to meet inclusion criteria, but because their contents could not be evaluated.

### 2.4 Data extraction

**Figure 1:**
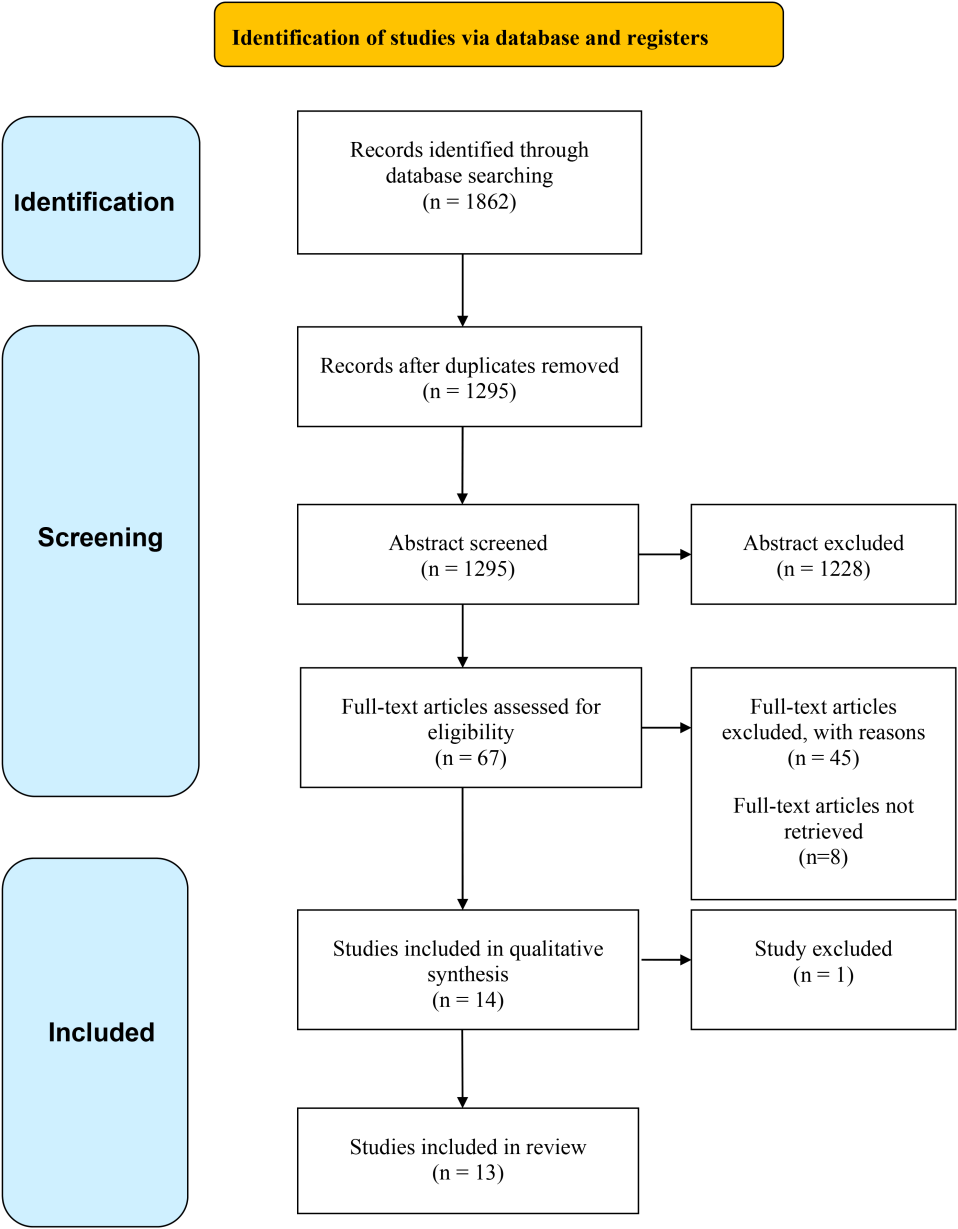
Prisma flowchart.

The focus of this review was on the sociodemographic and psychological characteristics of school shooters. Accordingly, all relevant variables addressed by the authors in the included studies were systematically extracted for analysis. The detailed study selection process is visually summarized in the flow diagram shown in **Figure 1**. This scheme, based on the PRISMA guidelines (40), clearly illustrates the number of records identified, screened, excluded, and ultimately included in the review.

All sociodemographic, psychological, behavioral, and experiential characteristics reported in the included studies were systematically extracted and organized into five thematic categories (**Tables 1**–**5**). **Supplementary Table S1** provides a comprehensive overview of how each characteristic was operationally defined across studies.

**Table 1:** Demographic characteristics of school shooters.

Characteristics	Dagenhard et al., 2019 (41)	Dowdell et al., 2022 (14)	Farr, 2019 (42)	Farr, 2018 (43)	Freilich et al., 2022 (1)	Freilich et al., 2022 (1)	Hall et al., 2019 (44)	Kowalski et al., 2021 (16)	Kowalski et al., 2021 (16)	Langman, 2016 (45)	Langman, 2009 (32)	Leary et al., 2003 (26)	Lenhardt et al., 2018 (46)	Paradice, 2017 (47)	Winch et al., 2024 (48)	Mean (available data)
Gender	25/25 (100%) Male	25/25 (100%) Male	N/A	N/A	247/253 (98%) Male 6/253 (2%) Female	97/101 (96%) Male 4/101 (4%) Female	47/49 (96%) Male 2/49 (4%) Female	54/57 (95%) Male 3/57 (5%) Female	23/24 (96%) Male 1/24 (4%) Female	61/64 (95%) Male 3/63 (5%) Female	10/10 (100%) Male	15/16 (94%) Male 1/16 (6%) Female	18/18 (100%) Male	334/343 (97%) Male 9/343 (3%) Female	N/A N/A	97% Male 3% Female
Age	N/A	12-26 Mean = 16.9	11-20 Mean =15.4	11-20 Mean = 15.4	Mean = 16.2	Mean = 33.8	12-62 Mean = 23.0	12-32 Mean = 15.0	11-62 Mean = 23.3	11-23 Mean = 15.7	11-23 Mean = 15.7	8-18 Mean = 14.3	N/A	6-70	Age range N/A Mean = 22.1	19.4
Ethnicity	N/A	N/A	N/A	25/31 (81%) White 4/31 (13%) Native American 2/31 (6%) Hispanic	64/253 (26%) White 135/253 (53%) Black 21/253 (8%) Hispanic 13/253 (5%) Asian 20/253 (8%) Other	24/69 (35%) White 31/69 (45%) Black 11/69 (16%) Hispanic 3/69 (4%) Other	N/A	35/57 (61%) White	8/24 (33%) White 9/24 (38%) Black 2/24 (8%) Asian	35/64 (55%) White 11/64 (17%) Black 8/64 (12%) Asian 5/64 (8%) Hispanic 4/64 (6 %) Native American 1/64 (2%) Middle Eastern	N/A	N/A	18/18 (100%) White	N/A	N/A	56% White 40% Black 10% Asian 9% Native American 8% Hispanic 4% Other
Declared sexual orientation	N/A	N/A	N/A	30/31 (98%) Heterosexual 1/31 (2%) Homosexual	N/A	N/A	N/A	N/A	N/A	N/A	N/A	N/A	N/A	N/A	N/A	98% Heterosexual 2% Homosexual

N/A indicates that the information was not available in this study.

**Table 2:** Social and relational experiences of school shooters.

Characteristics and authors	Dagenhard et al., 2019 (41)	Dowdell et al., 2022 (14)	Farr, 2019 (42)	Farr, 2018 (43)	Freilich et al., 2022 (1)	Freilich et al., 2022 (1)	Hall et al., 2019 (44)	Kowalski et al., 2021 (16)	Kowalski et al., 2021 (16)	Langman, 2016 (45)	Langman, 2009 (32)	Leary et al., 2003 (26)	Lenhardt et al., 2018 (46)	Paradice, 2017 (47)	Winch et al., 2024 (48)	Mean (available data)
Victim of bullying	11/19 (53%)	15/25 (60%)	N/A	10/31	73/253	N/A	N/A	25/57 (44%)	9/24 (37%)	N/A	N/A	12/16 (75%)	12/18 (67%)	N/A	N/A	50%
Use of social media	N/A	22/25 (88%)	N/A	N/A	N/A	N/A	N/A	N/A	N/A	N/A	N/A	N/A	N/A	N/A	N/A	88%
Romantic rejection	N/A	N/A	15/29 (52%)	30/31 (97%)	N/A	N/A	N/A	19/57 (33%)	5/24 (21%)	N/A	N/A	7/16 (44%)	11/18 (61%)	N/A	N/A	42%
Anger regulation difficulties	N/A	N/A	28/29 (97%)	N/A	N/A	N/A	N/A	N/A	N/A	N/A	N/A	N/A	11/18 (61%)	N/A	N/A	85%
Relational difficulties with females	N/A	N/A	17/31 (55%)	N/A	N/A	N/A	N/A	N/A	N/A	N/A	N/A	1/16 (6%)	N/A	N/A	N/A	31%
Negative peer labeling	N/A	N/A	23/31 (74%)	N/A	N/A	N/A	N/A	N/A	N/A	N/A	N/A	N/A	N/A	N/A	N/A	74%
Sense of isolation	N/A	N/A	22/31 (71%)	N/A	N/A	N/A	N/A	N/A	N/A	N/A	N/A	N/A	N/A	N/A	N/A	71%
Conflictual parental relationship	N/A	N/A	N/A	N/A	N/A	N/A	N/A	N/A	N/A	N/A	N/A	N/A	13/18 (72%)	N/A	N/A	72%
Grievance	N/A	N/A	N/A	N/A	N/A	N/A	N/A	N/A	N/A	N/A	N/A	N/A	N/A	N/A	19/80 (25%)	25%

N/A indicates that the information was not available in this study. Operational definitions of characteristics varied across studies. See **Supplementary Table S1** for detailed information on how each study defined and measured these variables.

**Table 3:** Mental and psychological health of school shooters.

Characteristics and authors	Dagenhard et al., 2019 (41)	Dowdell et al., 2022 (14)	Farr, 2019 (42)	Farr, 2018 (43)	Freilich et al., 2022 (1) Adolescents	Freilich et al., 2022 (1) Adults	Hall et al., 2019 (44)	Kowalski et al., 2021 (16) K-12	Kowalski et al., 2021 (16) College	Langman, 2016 (45)	Langman, 2009 (32)	Leary et al., 2003 (26)	Lenhardt et al., 2018 (46)	Paradice, 2017 (47)	Winch et al., 2024 (48)	Mean (available data)
Mental health disorders	6/25 (25%)	N/A	N/A	N/A	66/253 (26%)	27/101 (27%)	N/A	31/57 (54%)	16/24 (66%)	N/A	N/A	N/A	N/A	23/343 (7%)	N/A	34%
Psychiatric treatment preceding the attack	N/A	4/25 (16%)	N/A	16/31 (52%)	N/A	N/A	23/49 (47%)	N/A	N/A	N/A	N/A	1/16 (6%)	7/18 (39%)	N/A	N/A	38%
Mood disorders	N/A	5/25 (20%)	N/A	N/A	N/A	N/A	N/A	N/A	N/A	N/A	N/A	N/A	N/A	N/A	N/A	20%
Depression	N/A	N/A	25/29 (80%)	25/31 (81%)	N/A	N/A	8/49 (16%)	N/A	N/A	N/A	N/A	6/16 (37%)	11/18 61%)	N/A	N/A	55%
Neurodevelopmental disorders	N/A	N/A	N/A	N/A	N/A	N/A	3/49 (6%)	N/A	N/A	N/A	N/A	N/A	N/A	N/A	N/A	6%
Autism	N/A	4/25 (16%)	N/A	N/A	N/A	N/A	3/49 (6%)	N/A	N/A	N/A	N/A	N/A	N/A	N/A	N/A	11%
ADHD	N/A	2/25 (8%)	N/A	N/A	N/A	N/A	3/49 (6%)	N/A	N/A	N/A	N/A	1/16 (6%)	N/A	N/A	N/A	7%
Hallucinations/Delusions	N/A	N/A	N/A	13/31 (42%)	N/A	N/A	4/49 (8%)	N/A	N/A	N/A	5/10 (50%)	N/A	N/A	N/A	6/80 (8%)	27%
Schizophrenia or schizoaffective disorder	N/A	N/A	N/A	N/A	N/A	N/A	4/49 (8%)	N/A	N/A	N/A	N/A	1/16 (6%)	N/A	N/A	N/A	7%
Personality disorders	N/A	N/A	N/A	N/A	N/A	N/A	2/49 (4%)	N/A	N/A	N/A	N/A	N/A	N/A	N/A	N/A	4%
Psychopathic traits	N/A	N/A	N/A	N/A	N/A	N/A	N/A	N/A	N/A	N/A	2/10 (20%)	N/A	13/18 (61%)	N/A	N/A	40%
Obsessive-compulsive disorder	N/A	N/A	N/A	N/A	N/A	N/A	1/49 (2%)	N/A	N/A	N/A	N/A	N/A	N/A	N/A	N/A	2%
Anxiety disorder	N/A	N/A	N/A	N/A	N/A	N/A	4/49 (8%)	N/A	N/A	N/A	N/A	N/A	N/A	N/A	N/A	8%
Eating disorders	N/A	N/A	N/A	N/A	N/A	N/A	2/49 (4%)	N/A	N/A	N/A	N/A	N/A	N/A	N/A	N/A	4%
Substance use disorder	N/A	N/A	N/A	N/A	N/A	N/A	4/49 (8%)	N/A	N/A	N/A	N/A	1/16 (6%)	N/A	N/A	N/A	7%
Substance use disorder	N/A	N/A	N/A	N/A	N/A	N/A	4/49 (8%)	N/A	N/A	N/A	N/A	1/16 (6%)	N/A	N/A	N/A	7%
Neurodevelopmental disorders	N/A	N/A	N/A	N/A	N/A	N/A	3/49 (6%)	N/A	N/A	N/A	N/A	N/A	N/A	N/A	N/A	6%
Anxiety disorder	N/A	N/A	N/A	N/A	N/A	N/A	4/49 (8%)	N/A	N/A	N/A	N/A	N/A	N/A	N/A	N/A	8%
Psychopathic traits	N/A	N/A	N/A	N/A	N/A	N/A	N/A	N/A	N/A	N/A	2/10 (20%)	N/A	13/18 (61%)	N/A	N/A	40%

**Table 4:** Behavioral and risk factors of school shooters.

Characteristics and authors	Dagenhard et al., 2019 (41)	Dowdell et al., 2022 (14)	Farr, 2019 (42)	Farr, 2018 (43)	Freilich et al., 2022 (1)	Freilich et al., 2022 (1)	Hall et al., 2019 (44)	Kowalski et al., 2021 (16)	Kowalski et al., 2021 (16)	Langman, 2016 (45)	Langman, 2009 (32)	Leary et al., 2003 (26)	Lenhardt et al., 2018 (46)	Paradice, 2017 (47)	Winch et al., 2024 (48)	Mean (available data)
Communication about the impending attack	11/25 (46%)	18/25 (72%)	N/A	27/31 (87%)	N/A	N/A	N/A	N/A	N/A	N/A	N/A	N/A	N/A	N/A	N.A.	68%
Interest in violence	9/25 (38%)	N/A	N/A	15/31 (48%)	N/A	N/A	N/A	N/A	20/57 (35%)	8/24 (33%)	N/A	5/16 (31%)	8/18 (44%)	N/A	N/A	38%
Suicidal thoughts/attempts prior to the shooting	N/A	12/25 (48%)	22/29 (80%)	23/31 (74%)	N/A	N/A	N/A	N/A	N/A	N/A	N/A	3/16 (19%)	11/18 (61%)	N/A	39/80 (49%)	55%
Posting pictures of firearms	N/A	11/25 (44%)	N/A	N/A	N/A	N/A	N/A	N/A	N/A	N/A	N/A	N/A	N/A	N/A	N/A	44%
Posting threatening messages	N/A	10/25 (40%)	N/A	N/A	N/A	N/A	N/A	N/A	N/A	N/A	N/A	N/A	N/A	N/A	N/A	40%
Masculinity issues	N/A	N/A	N/A	31/31 (100%)	N/A	N/A	N/A	N/A	N/A	N/A	N/A	N/A	N/A	N/A	N.A.	100%
Academic/learning difficulties	N/A	N/A	N/A	12/31 (39%)	31/253 (12%)	N/A	N/A	N/A	N/A	N/A	N/A	N/A	N/A	N/A	N/A	25%
Interest/obsession with firearms	N/A	N/A	N/A	18/31 (58%)	N/A	N/A	N/A	10/57 (18%)	5/24 (21%)	N/A	2/10 (20%)	8/16 (50%)	N/A	N/A	N/A	33%
Cruelty to animals	N/A	N/A	N/A	N/A	N/A	N/A	N/A	N/A	N/A	N/A	N/A	3/16 (19%)	N/A	N/A	N/A	19%
Deficits in coping skills	N/A	N/A	N/A	N/A	N/A	N/A	N/A	N/A	N/A	N/A	N/A	N/A	15/18 (83%)	N/A	N/A	83%
Low resilience	N/A	N/A	N/A	N/A	N/A	N/A	N/A	N/A	N/A	N/A	N/A	N/A	17/18 (94%)	N/A	N/A	94%

N/A indicates that the information was not available in this study. Operational definitions of characteristics varied across studies. See **Supplementary Table S1** for detailed information on how each study defined and measured these variables.

**Table 5:** Traumatic experiences and health conditions of school shooters.

Characteristics and authors	Dagenhard et al., 2019 (41)	Dowdell et al., 2022 (14)	Farr, 2019 (42)	Farr, 2018 (43)	Freilich et al., 2022 (1) Adolescents	Freilich et al., 2022 (1) Adults	Hall et al., 2019 (44)	Kowalski et al., 2021 (16) K-12	Kowalski et al., 2021 (16) College	Langman, 2016 (45)	Langman, 2009 (32)	Leary et al., 2003 (26)	Lenhardt et al., 2018 (46)	Paradice, 2017 (47)	Winch et al., 2024 (48)	Mean (available data)
Violent or abusive domestic environment	6/25 (25%)	N/A	10/29 (33%)	13/31 (42%)	50/253 (20%)	30/101 (30%)	N/A	N/A	N/A	N/A	3/10 (33%)	N/A	N/A	15/343 (4%)	N/A	27%
At least one Adverse Childhood Experience	N/A	18/25 (78%)	N/A	N/A	N/A	N/A	N/A	N/A	N/A	N/A	N/A	N/A	N/A	N/A	N/A	78%
Physical health diagnosis	N/A	8/25 (32%)	N/A	N/A	N/A	N/A	N/A	N/A	N/A	N/A	N/A	N/A	N/A	N/A	N/A	32%

N/A indicates that the information was not available in this study. Operational definitions of characteristics varied across studies. See **Supplementary Table S1** for detailed information on how each study defined and measured these variables.

### 2.5 School shooting definitions in included studies

We included studies where authors explicitly addressed school shooting perpetrators, regardless of the specific definitional criteria employed by individual researchers. This inclusive approach was chosen to capture the full breadth of existing empirical research on the topic. As documented in **Table 6**, the included studies demonstrated considerable heterogeneity in their operational definitions and inclusion criteria. Definitions varied across multiple dimensions, including: the educational level of targeted institutions (K-12 vs. college/university), the requirement for casualties (ranging from “at least one injury” to specific victim counts), the shooter’s affiliation with the school (current vs. former students), the location of the incident (on school grounds vs. school-related events), and the temporal scope of data collection.

### 2.6 Ethical considerations regarding perpetrator identification

In accordance with established ethical guidelines and public health recommendations aimed at preventing media contagion effects, and consistent with the “No Notoriety” campaign principles (49, 50) this review deliberately avoids naming individual perpetrators. A substantial body of empirical evidence demonstrates that extensive media coverage naming and depicting mass shooting perpetrators can contribute to copycat phenomena and fame-seeking behavior among potential offenders (51–53).

Throughout this manuscript, we employ generic terminology (e.g., “school shooters,” “perpetrators”) and avoid identifying specific incidents or individuals beyond what is necessary for scientific discourse. When primary sources in our reference list contain location-specific incident names in their titles, these represent the original publications’ naming conventions and do not reflect our analytical approach. This methodology aligns with threat assessment best practices and minimizes potential glorification of perpetrators while maintaining the scientific integrity and replicability of our analysis. Individual cases examined in the included studies can be cross-referenced through the original publications’ methodological sections, which provide sufficient detail for independent verification and replication purposes.

## 3 Results

### 3.1 Included articles

The screening process led to the inclusion and analysis of *n* = 13 articles that met all inclusion criteria. **Table 6** presents the main characteristics of each study, including the authors and year of publication, study objective, methodology, sample size, data collection procedures, inclusion criteria, and time frame considered.

**Table 6:** Overview of the main features of the articles.

First author and year published	Study Objective	Study Type	Sample	Method & Data Collection	Inclusion Criteria	Time Frame
Dagenhard et al., 2019 (41)	Identify similarities among school shooting episodes.	Qualitative	*N* = 25 shooters	Examination of governors’ reports, police reports, and supporting court documents.	Must be a public K-12 school; must be an intentional mass homicide; at least one victim; occurred on school premises.	1999-2014
Dowdell et al., 2022(14)	Report media-published information on major risk factors linked to school attackers and their use of social media.	Quantitative	*N* = 25 male shooters	Critical and systematic review of national and local media reports.	Shootings in middle school, high school, or college campuses; aged 12–26; shooters current or former students of the targeted school.	2013–2019
Farr, 2019(42)	Consider the role of romantic rejection in violent school shootings by adolescent males.	Qualitative	*N* = 29 male shooters	Consultation of websites providing materials on school shootings (articles, reports, trial transcripts).	Shooting in elementary, middle, or high school; perpetrator a current or former student under age 21; attack occurred at school, schoolyard, or during a school event; shooter fired at two or more people, at least one being a student, or at a group gathering affiliated with the school including students; at least one person shot was not a specifically targeted victim.	1995–2015
Farr, 2018(43)	Examine the link between fragile masculinity and acts of violence committed by adolescents in schools.	Qualitative	*N* = 31 male shooters	Review of multiple lists of school shootings in primary and secondary schools to select shooters meeting inclusion criteria.	Attacked school was elementary, middle, or high school; perpetrator a current or former student under age 21; attack occurred at school, schoolyard, or school event; shooter fired at two or more people, at least one being a student, or at a group/gathering including at least one student; at least one person shot was not a specifically targeted victim.	1995–2015
Freilich et al., 2022 (1)	Use open-source data to study school gun violence incidents in the U.S. and build a national database to fill current research gaps.	Quantitative	*N* = 354 shooters: 253 adolescents 101 adults (cut-off age = 20)	Examination of 40+ sources, including existing databases, timelines/lists, official records, law enforcement reports, academic works, media lists, online encyclopedias, blogs, and advocacy reports.	Shooting occurred in the U.S.; generated a criminal justice response; firearm involved; occurred in a K-12 school; at least one person injured or killed.	1990–2016
Hall et al., 2019 (44)	Determine the percentage of individuals involved in school shootings who had previously taken psychotropic medications.	Qualitative	*N* = 49 shooters	Retrospective analysis of school shooting perpetrators using publicly accessible records.	Incidents occurring on school campuses during times when students/educators were present or expected; shootings on schoolyards or school grounds.	2000–2017
Kowalski et al., 2021 (16)	Compare variables associated with three types of armed violence: K-12 shootings, college/university shootings, and mass shootings in general.	Quantitative	*N* = 57 K-12 shooters; *N* = 24 college shooters.	Comparison of existing lists of K-12 and college shootings with inclusion criteria.	K-12: occurred at school during school hours; perpetrator current/former student; shootings in kindergarten–12th grade schools. College: shootings occurred on campus or at school-sponsored off-campus events; perpetrator current/former student; at least one person other than the shooter (not necessarily a student) killed or injured.	2001–2018
Langman, 2016 (45)	Expand literature by examining changes in techniques used by U.S. school shooters over 50 years.	Qualitative	*N* = 64 shooters	Analysis of academic works listing/profiling school shooters, plus online lists/databases.	Attacks occurred in educational contexts; involved firearms; premeditated; caused ≥3 victims injured or killed (excluding shooters themselves or police); not due to gang violence; not intimate partner violence within school property; occurred in U.S.	1966–2015
Langman, 2009 (32)	Highlight major differences among school shooters by presenting a three-category typology.	Qualitative	*N* = 10 shooters	Case research emphasizing available pre-shooting information.	Shooters selected for data richness and information consistency.	1997–2007
Leary et al., 2003 (26)	Examine the role of social rejection in school violence.	Qualitative	*N* = 16 shooters	Analysis of well-documented U.S. school violence cases from national media, newspapers, and websites.	Must have occurred during school hours; perpetrated by students; caused injury/death of at least one student; incidents where only non-students were victims excluded.	1995–2001
Lenhardt et al., 2018 (46)	Report findings from an analysis of 18 school shooters.	Qualitative	*N* = 18 shooters	Review of archival sources (≥12 per case) and inclusion criteria	Only secondary school shootings in the U.S.; premeditated/planned attacks on a school.	1996–2012
Paradice, 2017 (47)	Describe the construction and detailed analysis of a U.S. school shooting dataset.	Qualitative	*N* = 343 events	Analysis of Wikipedia entry “List of School Shootings in the U.S.” and linked sources; compared with inclusion criteria.	Event included if firearm used in a school or on school grounds, regardless of casualties.	1840–2015
Winch et al., 2024 (48)	Build on prior studies to identify variables distinguishing completed vs. averted school shootings.	Quantitative	*N* = 229 cases: 149 averted 80 completed – sample of interest	Cases drawn from Averted School Violence database; analysis of related news articles, Google searches, and, when possible, information from involved parties; statistical analysis conducted to compare groups	Schoolyard shootings (elementary, secondary, college); involved/planned firearm use; not linked to organized crime; labeled as completed if occurred on school grounds or school event and caused ≥1 injury; labeled as averted if evidence of preparatory steps (e.g., researching prior shootings, gun purchase, drafting attack plan).	1999–2020

The 13 included studies demonstrated considerable methodological diversity in their approaches to investigating school shooter characteristics. The sample encompassed both qualitative (*n* = 8) and quantitative (*n* = 5) research designs, with substantial variation in sample sizes ranging from 10 to 354 cases. Studies differed significantly in their temporal scope, with some focusing on narrow time windows (e.g., 14: 2013-2019) while others spanned several decades (e.g., 1: 1990-2016; 47: 1840-2015). The research objectives varied considerably, encompassing typological analyses (32), gender-specific investigations (42, 43), comparative studies across different educational levels (16), and database construction efforts (1, 47). Data collection methods also showed marked heterogeneity, including analysis of official documents and court records (41), media reports (14), archival sources (46), existing databases (48), and online resources (42, 43). The populations of school shooters examined across studies were not independent. There was likely considerable overlap between studies, meaning that individual shooters may have appeared in multiple datasets.

Moreover, it became evident that different authors employed distinct inclusion criteria, focusing on diverse aspects of the school shooting phenomenon. Despite this heterogeneity, none of the studies explicitly set out to examine in depth the sociodemographic and psychological characteristics of school shooters as their primary objective. Nonetheless, all of them addressed certain relevant features, such as personality traits, family and social background, psychological distress or marginalization, as well as possible instigating factors and warning signs that may have foreshadowed the escalation into violence.

### 3.2 Collected data

**Tables 1**–**5** summarize the frequencies of all sociodemographic and psychological characteristics of school shooters identified in the included articles. Each cell presents both the number of individuals (*n*) displaying the corresponding characteristic and the percentage (in parentheses) relative to the total sample. The division of characteristics into tables follows a thematic categorization, resulting in the following groups: demographic/personal data, social and relational experiences, mental and psychological health, risk behaviors/factors, and traumatic experiences. For the last column of each table, average values were reported. These represent mean frequencies, except for the variable “Age,” where the average of the means was calculated. In cases where data were unavailable for any reason, the notation “N/A” (Not Available) was applied.

The sociodemographic characteristics of school shooters in the United States are summarized in **Table 1**. Recurrent patterns emerge with respect to gender, age, and ethnic background, which help outline the predominant sociodemographic profile of the perpetrators. The data highlight concentrations within specific population groups, offering an important basis for understanding the broader social and cultural contexts in which these events took place.

The social and relational experiences of the individuals analyzed are presented in **Table 2**, with particular attention to family dynamics, school experiences, and peer relationships. The data frequently point to conflictual family interactions, social isolation, and difficulties integrating within peer groups. Experiences of bullying, marginalization, or family instability emerge as recurrent themes, suggesting a background of vulnerability that may interact with other risk factors.

The mental and psychological health conditions associated with school shooters are documented in **Table 3**. The results show a high prevalence of psychopathological symptoms, ranging from depression and anxiety to more structured psychiatric diagnoses, along with reports of suicidal ideation.

Behavioral patterns and commonly reported risk factors—such as substance use, access to firearms, episodes of violent conduct, and behavioral warning signs—are highlighted in **Table 4**. These elements are crucial for understanding the progression toward violent acts, as they point to observable indicators that can serve as early warning signs for preventive intervention.

Data on the traumatic experiences and physical health conditions of school shooters are presented in **Table 5**. Many individuals displayed histories of abuse, neglect, or other significant traumatic events, often intertwined with chronic or poor health conditions. These factors contribute to a profile in which trauma and health difficulties overlap with additional vulnerabilities, thereby amplifying the risk of extreme violent behaviors.

## 4 Discussion

### 4.1 Considerations and suggestions for future research

**Table 1** highlights a clear predominance of male participants, who represent an average of 97% of the samples, compared to a minimal female presence of only 3%. The pronounced gender disparity observed is consistent with broader patterns of firearm-related violence (54) and should therefore be interpreted as part of these wider trends rather than as a distinctive feature of school shootings. Regarding age, the samples primarily consisted of younger individuals, with an overall mean age of 19.4 years. The reviewed literature showed that most studies focused on adolescent and young adult school shooters; however, one study reported an older mean perpetrator age of 33.8 years. From an ethnicity perspective, a certain degree of heterogeneity was observed. White participants constituted an average of 56%, followed by Black or African American participants at 40%. Other ethnic groups, such as Asian, Hispanic, and other minorities, appeared in smaller proportions ranging from 4% to 10%. However, several studies did not report ethnicity.

Across **Tables 1**–**5**, the most striking finding is the predominance of missing data. The absence of information on the characteristics of school shooters represents the main outcome of this study. Out of 645 cells, 482 were labeled “N/A,” accounting for 75% of the dataset. This scarcity inevitably affects the calculated averages, as many characteristics are represented by only one or a few data points. Of the 42 characteristics considered, 26 are represented by one or two values at most, corresponding to 60% of the total. Consequently, the representativeness of these averages cannot be determined, as it is not possible to conduct analyses of standard deviations that would clarify the extent to which results deviate from overall means. The tables also suggest that school shooters are not a homogeneous group: demographic, experiential, psychological, relational, and familial differences indicate the likely existence of multiple underlying profiles. However, testing this hypothesis would require quantitative analyses capable of combining variables, which is currently hindered by the lack of data. The overlapping of samples, already noted in Section 3.1, further reduces the value of the figures presented in these tables.

In light of these considerations, a possible direction for future research would be to address the issue of overlapping samples. A second review of subject lists, where available, could allow the reconstruction of a new dataset in which each shooter is counted only once. This would produce more precise and representative data, avoiding duplications that may bias the analysis. Another important step would be to fill existing information gaps by conducting in-depth investigations of individual cases included in this review. Drawing also on media sources, this approach could recover missing data and supplement the findings summarized in **Tables 1** through 6, which currently serve as useful but incomplete reference points.

The scarcity of data in the tables likely reflects a broader issue in the existing literature, which remains fragmented and sparse regarding the characteristics of school shooters. Moreover, an important factor contributing to this scarcity involves the legal constraints on accessing information about juvenile offenders. Since a substantial proportion of school shooters are under 18 years of age (as shown in **Table 1**, with mean ages ranging from 14.3 to 23 years across studies), researchers face significant barriers in obtaining comprehensive data. In the United States, juvenile criminal records are confidential by law in all states unless the minor is tried as an adult (transferred to adult court). This legal protection of juvenile records, while serving important privacy and rehabilitation purposes, creates structural obstacles to systematic data collection on school shooter characteristics. Consequently, detailed psychological, psychiatric, educational, and family background information may remain inaccessible to researchers, even when such information exists in official records. This legal barrier represents a fundamental challenge that cannot be overcome simply through improved research protocols, and must be acknowledged as an inherent limitation in this field of study.

This significantly hinders data collection and analysis. The tables presented in Section 3.2 depict the current state of factors that researchers deem important in studying school shooters. However, the lack of a standardized protocol for data collection prevents critical interpretation and deeper analysis. On this basis, it can be hypothesized that introducing a standardized data collection protocol into research methodology on school shooters would substantially advance the field. Such standardization would provide a shared knowledge base, facilitating dialogue and collaboration among research groups. Moreover, it would allow for easier identification of patterns or correlations across characteristics, promoting a more comprehensive understanding of the phenomenon. A concrete recommendation for future studies would be the inclusion, at the end of each study, of a final table listing the shooters in the sample together with the characteristics examined, as outlined in Section 3.2, in order to provide subsequent researchers with comparable and combinable datasets. However, the feasibility of this recommendation depends on the legal constraints governing the disclosure of identifying information—particularly stringent when minors are involved. Researchers should therefore first verify whether the applicable legislation permits the publication of such data and, if necessary, adopt anonymized or partially de-identified formats that ensure compliance with privacy and juvenile protection laws. Beyond legal requirements, future research should adopt the ethical frameworks discussed in Section 2.6, employing anonymized case identification systems that enable cross-study data integration without contributing to perpetrator notoriety. The development of coordinated anonymization protocols—such as standardized alphanumeric coding systems shared across research groups—would facilitate systematic data collection while adhering to public health best practices.

### 4.2 Limitations

Although one methodological challenge has already been discussed in Section 4.1 (the overlapping of samples), this study is not exempt from additional limitations. While the primary purpose of a systematic literature review is to cover the entirety of the material on the topic of interest, certain publications may not have been included in this research, as they may be retrievable only through additional databases.

Another limitation concerns the lack of standardized definitions for the characteristics reported in the tables. This study did not explicitly clarify each variable (for example, what is precisely meant by Adverse Childhood Experiences and how these are distinguished from other negative experiences). As a result, the objectivity of how each characteristic is conceived or perceived cannot be assumed (see **Supplementary Table S1** for detailed comparisons).

It should also be noted that many school shooter die by suicide at the time of the violent event (55). This represents a further complication for data collection, as it prevents direct access to information from the source itself. Moreover, most of the studies rely on relatively small samples of U.S. school shooters.

Additionally, legal protections surrounding juvenile criminal records in the United States create structural barriers to accessing comprehensive information about school shooters under the age of 18, who constitute a significant proportion of perpetrators. Moreover, most of the studies rely on relatively small samples of U.S. school shooters.

### 4.3 Strengths

Despite the limitations discussed above, the present study also has several strengths. First, it represents, to our knowledge, the first systematic review specifically focused on the socio-demographic and psychological characteristics of school shooters in the United States. Second, the systematic organization of the extracted data into thematic tables (demographic characteristics, social and relational experiences, mental health and psychological conditions, risk behaviors, and traumatic experiences) provides an accessible and structured framework. These tables can serve as reference points for future research and facilitate the identification of gaps in the existing literature. Finally, by explicitly highlighting the predominance of missing data, the study makes a significant contribution by underscoring a critical issue in current scholarship. The identification of this gap not only informs future research directions but also emphasizes the urgent need for standardized protocols in the collection and reporting of data on school shooters.

## 5 Conclusions

This literature review ultimately revealed the scarcity and fragmentation of existing data on the socio-demographic and psychological characteristics of school shooters. This finding provides a realistic depiction of the current state of research in the field and offers valuable guidance for future directions. Indeed, awareness of this knowledge gap represents a crucial starting point for advancing the understanding of school shootings.

On the basis of these considerations, it is not possible to identify a definitive profile—or even multiple profiles—of school shooters, as noted in the introduction to this study. Although this review does not propose the existence of a standardized “average” perpetrator, any potential recurring patterns of sociodemographic and psychological characteristics among school shooters remain impossible to delineate due to the insufficiency and fragmentation of the data collected thus far. The absence of clearly identifiable common characteristics may not necessarily reflect the nonexistence of meaningful patterns, but rather the inadequate and fragmented nature of existing data collection and reporting practices, which have prevented such patterns from emerging with sufficient clarity and statistical reliability.

This review aims to stimulate researchers to systematize empirical investigations of school shooter characteristics. The findings presented here underscore the critical need for standardized data collection protocols, consistent definitional frameworks, and comprehensive reporting of school shooter characteristics. These methodological advancements would foster a deeper understanding of the phenomenon.

## Data Availability

The original contributions presented in the study are included in the article/**Supplementary Material**. Further inquiries can be directed to the corresponding author.
