# Supplementary material for: Sociodemographic and psychological characteristics of school shooters in the United States: a systematic review of the literature
Source: Front Psychiatry. 2026 Jan 12;16:1735929. doi: 10.3389/fpsyt.2025.1735929 (PMC12833454; doi:10.3389/fpsyt.2025.1735929)
Supplement: Supplementary file 1 [file Table1.docx]

SUPPLEMENTARY TABLE S1

Operational Definitions of Characteristics Across Included Studies

| SOCIAL AND RELATIONAL EXPERIENCES | Key Operational Details Across Studies |
| --- | --- |
| Victim of bullying | Dagenhard et al. (2019): Coded when case records or the perpetrator’s own statements documented *severe bullying in school by peers*, i.e. sustained, significant peer harassment prior to the attack.  Dowdell et al. (2022): Bullying was defined (following CHOP) as intentional, mean, aggressive behavior that is *repeated over time* and occurs in the context of an actual or perceived power imbalance, including physical, verbal, relational, or cyber forms; a case was coded positive when open-source reports indicated the shooter had experienced in-person or online bullying.  Farr (2018): Victimization by bullying was inferred when sources described the shooter as subjected to *emasculating bullying and intentional marginalization by male peers* (e.g. persistent taunts, homo-epithets, and humiliating, often sexualized physical victimization) that exposed failures in Adolescent Insider Masculinity.  Freilich et al. (2022): The closest construct is the TASSS variable *“experienced peer aggression”*, coded present when open-source information showed that the perpetrator had been involuntarily and personally victimized by aggressive acts from non-familial peers (e.g. verbal threats/coercion or physical attacks); mere teasing without clear aggression was not coded.  Kowalski et al. (2021): A documented pattern of chronic ostracism, bullying, or malicious teasing—defined as verbal or physical abuse intended to harm or injure.  Leary et al. (2003): Social rejection via bullying was coded when reports indicated an *ongoing pattern of malicious teasing or bullying* (e.g. being regularly taunted, publicly humiliated, called “nerd,” “dweeb,” “faggot,” or otherwise picked on) and/or chronic ostracism that placed the perpetrator on the periphery of school social life.  Lenhardt et al. (2018): The individual-factor item *“alienation/bullied”* was coded when case materials showed that the shooter felt alienated, had been bullied, or both, typically in the context of increased bullying, harassment, and marginalization that contributed to a sense of alienation and persecution. |
| Use of social media | Dowdell et al. (2022): Defined as the presence, activity, and behavioral patterns on shooters’ social media accounts, identified through open-source digital traces. Coding focused on whether offenders *used social media platforms* (e.g., Facebook, Twitter, Instagram, Snapchat) and how these spaces were *utilized to express grievances, hostility, violent ideation, or leakage*. The variable was not limited to frequency but included *qualitative indicators of maladaptive online engagement* (e.g., posting violent content, threats, or hostile statements). |
| Romantic rejection | Farr (2019): Defined as a verifiable romantic breakup or failure to attract a desired girl that occurred in the months prior to the shooting.  Farr (2018): Defined as failed or thwarted romantic/sexual pursuits that resulted in emotional humiliation.  Kowalski et al. (2021): Romantic rejection was subsumed under a broader construct of “history of rejection”, which included failures in romantic or sexual relationships.  Leary et al. (2003): Operationalized as romantic/social rejection that involved actual dismissals or perceived exclusion by desired partners, including being turned down, socially ignored by girls, or humiliated in romantic contexts.  Lenhardt et al. (2018): The closest relevant construct was “romantic struggles” within the domain of individual-level stressors, coded when case materials showed: distress linked to breakups, feelings of romantic inadequacy, unreciprocated romantic interest. |
| Anger regulation difficulties | Farr (2019): Anger regulation difficulties are understood as persistent struggles to manage, modulate, or express anger in socially appropriate ways.  Lenhardt et al. (2018): difficulties with anger were coded when the offender demonstrated recurrent problems controlling emotional arousal, such as disproportionate anger responses, impulsive outbursts, or chronic hostility toward peers or authority figures. |
| Relational difficulties with females | Farr (2018): Farr conceptualizes relational difficulties with females as a pattern of strained, conflictual, or humiliating interactions with girls or young women.  Leary et al. (2003): conflictual experiences with females were coded when journalistic or investigative sources documented that the perpetrator felt rejected, belittled, ignored, or publicly embarrassed by girls, or perceived chronic failure in gaining their approval or attention. |
| Negative peer labeling | Farr (2018): Negative peer labeling refers to the assignment of derogatory, emasculating, or stigmatizing labels by peers, typically male classmates. These labels often framed the perpetrator as weak, strange, sexually inadequate, or socially irrelevant.  Leary et al. (2003): negative peer labeling is understood as repeated assignment of demeaning or humiliating names, including insults that target the perpetrator’s social status, appearance, sexual identity, or perceived weaknesses. These labels were coded when credible reports indicated that shooters were frequently called derogatory names such as “nerd,” “freak,” “loser”. |
| Sense of isolation | Farr (2018): Farr interprets the shooter’s sense of isolation as a pervasive perception of being socially peripheral, unseen, or excluded within adolescent peer culture. |
| Conflictual parental relationship | Lenhardt et al. (2018): In Lenhardt’s framework, conflictual parental relationships are understood as patterns of chronic tension, hostility, or dysfunctional interaction between the shooter and one or both parents, reflected in case materials that documented frequent arguments, punitive or inconsistent parenting, emotional distance, or overt family conflict. |
| Grievance | Winch et al. (2024): Any form of grievance, whether it be towards a member of school personnel, students, or just people in general. Cases in which a person had a specific “hit list” are included here. Additionally, in this category, there are cases that use phrases “hating everyone at the school” in reference to people not based on race or religious affiliation |
| MENTAL AND PSYCHOLOGICAL HEALTH |  |
| ADHD | Dowdell et al. (2022): they code the variable as *present* when news reports describe a prior diagnosis of attention-deficit/hyperactivity disorder. This means ADHD is conceptualized primarily as a historical diagnostic label assigned by external sources, rather than as a behavioral construct operationalized through symptoms.  Hall et al. (2019): Hall operationalizes ADHD as one of several formal psychiatric diagnoses documented in the medical, legal, or investigative records of the shooters.  Leary et al. (2003): ADHD is mentioned in the case descriptions when a perpetrator had a documented history of attention-deficit/hyperactivity disorder. As in the other studies, ADHD is not defined behaviorally but appears as a background psychiatric condition. |
| Psychiatric treatment preceding the attack | Dowdell et al. (2022): psychiatric treatment preceding the attack is coded whenever open-source reports indicate that the shooter had received mental health services or psychiatric care before the incident. The definition is broad and includes any documented contact with mental health professionals, use of counseling services, or engagement in treatment pathways, regardless of duration or intensity. The variable reflects the presence of prior mental health intervention rather than its clinical content.  Farr (2018): Farr refers to psychiatric treatment only when cases document a clear history of mental health care that predates the attack, including past therapy, psychiatric hospitalization, or ongoing treatment.  Hall et al. (2019): Hall conceptualizes psychiatric treatment preceding the attack as any verified form of mental health intervention occurring before the shooting. This includes psychotherapy, medication management, psychiatric evaluation, or hospitalization. The coding is based on medical or legal documentation.  Leary et al. (2003): Leary includes psychiatric treatment when investigative or journalistic sources indicate that a perpetrator had previously received therapy, counseling, or psychiatric services.  Lenhardt et al. (2018): Lenhardt codes psychiatric treatment as part of the individual-level factors when case materials demonstrate a documented history of mental health services prior to the shooting. This includes counseling, psychiatric evaluation, prescribed medication, or other mental health interventions. |
| Depression | Farr (2019): depression is examined as part of the broader socio-emotional landscape that shapes adolescent masculine identity. It is operationalized implicitly when case materials describe persistent sadness, hopelessness, withdrawal, or emotional collapse following experiences of rejection, humiliation, or social marginalization.  Farr (2018): Depression is conceptualized as a recurring affective state linked to failed attempts at achieving masculine recognition. It is operationalized when case narratives depict chronic low mood, despair, or emotional shutdown as a response to bullying, romantic failure, or exclusion.  Hall et al. (2019): Hall codes depression as a documented psychiatric diagnosis identified through medical records, legal files, or investigative reports. It is treated as a formal mental health condition, not inferred from behavior, and appears in the analysis when the shooter had previously been diagnosed with major depression or another depressive disorder.  Leary et al. (2003): In Leary’s framework, depression is referenced only when explicitly mentioned in credible media or investigative sources. The variable is not formally operationalized but is treated as a background psychological characteristic, included when reports indicate that the perpetrator had a history of depression, exhibited longstanding sadness, or received treatment for depressive symptoms.  Lenhardt et al. (2018): Depression is coded when case documentation shows either a prior clinical diagnosis or clear evidence of depressive symptomatology (such as persistent low mood, withdrawal, or hopelessness). |
| Autism | Dowdell et al. (2022): Autism as a reported developmental or psychiatric diagnosis extracted from open-source media accounts. The study does not operationalize autism behaviorally, nor does it differentiate among autism spectrum subtypes. Instead, autism is coded as present when journalistic sources explicitly state that the perpetrator had been diagnosed with an autism spectrum disorder.  Hall et al. (2019): Hall provides the most structured operationalization of autism among your included studies. Autism is coded when medical or psychological records indicate a formal diagnosis of autism spectrum disorder (ASD) or when clinical evaluations document autistic traits consistent with ASD criteria. The article treats autism as a clinically verified developmental disorder. |
| Mood disorders | Dowdell et al. (2022): mood disorders are treated as reported clinical diagnoses drawn from open-source media accounts. Mood disorders are conceptualized as historical diagnostic labels. |
| Hallucinations/Delusions | Farr (2018): Farr does not conceptualize hallucinations or delusions as clinical constructs to be diagnosed; instead, they appear in her analysis only when case narratives describe shooters exhibiting psychotic-like experiences, such as hearing voices or holding fixed, irrational beliefs that depart from consensual reality.  Hall et al. (2019): These symptoms are coded only when clinical records, psychiatric evaluations, or official investigative documents confirm the presence of psychotic features such as auditory hallucinations, persecutory delusions, or other disturbances in thought content. The authors distinguish psychotic symptoms from other mental disorders and treat them as clinically verified indicators of psychosis.  Langman (2009): He identifies these symptoms when case evidence shows clear breaks from reality, such as the shooter believing others were plotting against him, experiencing command hallucinations, or maintaining paranoid or grandiose delusional systems.  Winch et al. (2024): These psychotic features are included when official reports indicate that the individual experienced distorted or false perceptions or beliefs, such as hearing voices, misinterpreting reality, or holding fixed delusional ideas. The construct is treated as a behaviorally observable and formally documented risk marker. |
| Mental health disorders (general) | Dagenhard et al. (2019): Dagenhard conceptualizes mental health disorders as any documented psychological or psychiatric condition reported in official investigative material, clinical evaluations, or credible secondary sources. The construct is intentionally broad and includes mood disorders, anxiety disorders, psychotic disorders, and behavioral or developmental conditions when such diagnoses were explicitly stated in the records reviewed.  Freilich et al. (2022): The definition includes both DSM-based disorders (e.g., depression, anxiety, psychotic disorders) and broader categories such as behavioral or emotional disorders when these are formally identified.  Kowalski et al. (2021): Disorders are coded when published investigative reports indicate the presence of a clinically diagnosed mental illness, such as depression, bipolar disorder, anxiety disorders, or psychosis. |
| Schizophrenia or schizoaffective disorder | Dagenhard et al. (2019): Dagenhard conceptualizes schizophrenia and schizoaffective disorder as clinically documented psychotic disorders, included when official investigative files or credible clinical records explicitly identify one of these diagnoses.  Freilich et al. (2022): Freilich and colleagues adopt a strictly evidence-based operationalization. Schizophrenia and schizoaffective disorder are coded within the TASSS dataset only when open-source records contain explicit confirmation of such diagnoses. The distinction between adolescents and adults does not alter the coding rule: the diagnosis must be explicitly named.  Kowalski et al. (2021): Kowalski treats schizophrenia and related psychotic disorders as part of a broader domain of clinically diagnosed mental illness, coded when investigative, legal, or medical sources specify that the perpetrator had been diagnosed with schizophrenia, schizoaffective disorder, or another psychotic condition.  Paradice (2017): Paradice does not operationalize schizophrenia or schizoaffective disorder as discrete coded variables. However, the dataset includes cases where shooters were described in historical accounts as having “serious mental illness,” “psychosis,” or “schizophrenia.” These references appear only when historical documents explicitly report such diagnoses. |
| Personality disorders | Hall et al. (2019): Hall operationalizes personality disorders as documented clinical diagnoses identified through medical, legal, or investigative records, rather than through behavioral inference. |
| Obsessive-Compulsive Disorder | Hall et al. (2019): Hall conceptualizes obsessive–compulsive disorder as a formally documented psychiatric diagnosis, identified exclusively through clinical, legal, or investigative records that explicitly state the presence of OCD in the shooter’s history. |
| Eating disorders | Hall et al. (2019): In Hall’s analysis, eating disorders are treated as formally documented psychiatric conditions, coded only when medical, psychological, or investigative records explicitly report that the perpetrator had been diagnosed with an eating disorder prior to the attack. |
| Substance use disorder | Hall et al. (2019): Hall conceptualizes substance use disorder as a formally documented diagnosis of problematic or disordered use of alcohol, illicit drugs, or misused prescription medications, identified through clinical, legal, or investigative records.  Leary et al. (2003): Leary does not treat substance use disorder as a clinical construct, nor does he use diagnostic terminology. However, the study includes substance use when case narratives explicitly report regular, problematic, or escalating consumption of alcohol or drugs that contributed to deterioration in functioning or increased psychosocial risk. |
| Neurodevelopmental disorders | Hall et al. (2019): Hall operationalizes neurodevelopmental disorders as clinically verified developmental conditions diagnosed during childhood or adolescence, based on medical or psychological records reviewed by the authors. |
| Anxiety disorder | Hall et al. (2019): Hall operationalizes anxiety disorder as a formally documented clinical diagnosis, identified through medical, psychiatric, or investigative records indicating that the perpetrator had been diagnosed with an anxiety-related condition. |
| Psychopathic traits | Langman (2009): Psychopathic traits are operationalized as a constellation of interpersonal, affective, and behavioral characteristics consistent with clinical descriptions of psychopathy, though not necessarily tied to a formal diagnosis.  Lenhardt et al. (2018): Although the study does not use formal psychopathy scales, it codes psychopathic traits when case documentation indicates persistent patterns of callousness, lack of remorse, manipulative social behavior, or chronic violation of social norms. |
| BEHAVIORAL AND RISK FACTORS |  |
| Communication about the impending attack | Dagenhard et al. (2019): Dagenhard operationalizes communication about the impending attack as any form of leakage, meaning intentional or unintentional disclosures, verbal or written, in which the perpetrator hinted at, threatened, or directly described plans for violence prior to the shooting.  Dowdell et al. (2022): communication about the impending attack is identified through open-source reporting that the shooter expressed violent intent or threatened harm before the event, either in person or through digital platforms.  Farr (2018): Farr addresses communication of impending violence within the broader narrative of masculinity, focusing on instances where shooters made boastful, provocative, or aggressive statements that partially revealed their intentions. |
| Interest in violence | Dagenhard et al. (2019): Dagenhard conceptualizes interest in violence as a documented fascination with violent content, weapons, or previous acts of mass or school violence, identified through pre-attack behaviors and statements.  Farr (2018): It refers to shooters’ engagement with violent fantasies, aggressive symbolism, or violent cultural material that served as emotional compensation or identity reinforcement.  Kowalski et al. (2021): Kowalski operationalizes interest in violence as documented engagement with violent entertainment, violent ideation, or weapon-related material, coded when investigative sources report that the perpetrator consumed violent media (such as violent films, games, or music), expressed enthusiasm for violent acts, or demonstrated a fascination with past shootings.  Leary et al. (2003): Leary includes interest in violence when case reports show that the shooter had expressed admiration for violent behavior, discussed violent fantasies, or consumed violent media in ways that appeared meaningfully connected to their personal grievances or emotional instability.  Lenhardt et al. (2018): The variable is coded when case documentation indicates persistent fascination with violent acts, weapons, or aggressive behavior, such as writing about violence, collecting violent imagery, or repeatedly discussing violent scenarios. |
| Suicidal thoughts/attempts prior to shooting | Dowdell et al. (2022): suicidal thoughts or attempts are coded when open-source media reports explicitly state that the perpetrator had expressed suicidal ideation or engaged in a suicide attempt prior to the attack.  Farr (2019): Farr conceptualizes suicidality within a broader narrative of gendered distress and emotional collapse, referencing suicidal thoughts or attempts when case narratives describe shooters expressing a desire to die, engaging in self-harm, or contemplating suicide in the context of perceived humiliation, social rejection, or compromised masculinity.  Farr (2018): suicidality is discussed when shooters showed self-destructive ideation or explicit contemplation of death, often linked to rejection, bullying, or perceived gendered inadequacy.  Leary et al. (2003): Leary includes suicidal thoughts and attempts when investigative reports indicate that the perpetrator had expressed self-destructive intent, engaged in previous suicidal behaviors, or communicated a desire to die prior to the shooting.  Lenhardt et al. (2018): Lenhardt codes suicidal ideation or attempts as an individual-level psychological risk factor, identified when case documentation provides evidence of prior suicidal thoughts, plans, gestures, or attempts.  Winch et al. (2024): Winch operationalizes suicidality within the threat-assessment framework as documented suicidal ideation, plans, or attempts recorded in school, police, or behavioral assessment files. |
| Posting pictures of firearms | Dowdell et al. (2022): In this study, “posting pictures of firearms” is treated as a specific digital behavior identified through open-source media reports, and is coded when the perpetrator had previously uploaded photographs or images displaying firearms on social media platforms. |
| Posting threatening messages | Dowdell et al. (2022): posting threatening messages is defined as the public dissemination of written or digital communications that express violent intent, threaten harm, or imply preparation for an attack, when such communications were shared on social media platforms and subsequently reported in open-source media accounts. |
| Masculinity issues | Farr (2018): masculinity issues refer to developmental struggles in meeting culturally enforced expectations of male adequacy, particularly in high-school social environments where status, heterosexual success, and physical or emotional toughness are heavily policed by peers. |
| Academic/learning difficulties | Farr (2018): These difficulties are referenced when case narratives show that the shooter experienced poor academic performance, struggles with schoolwork, or negative interactions with teachers related to academic failure.  Freilich et al. (2022): Academic and learning difficulties are operationalized through explicit references in open-source records to educational impairment, such as diagnosed learning disabilities, enrollment in special education programs, chronic academic underachievement, or documented struggles in meeting academic expectations. |
| Interest/obsession with firearms | Farr (2018): The construct includes behaviors such as regularly discussing guns, admiring weaponry, or expressing fascination with violent instrumental power.  Kowalski et al. (2021): Kowalski operationalizes interest in firearms as documented enthusiasm for guns, weapon ownership, or weapons-related behavior, coded when investigative reports confirm repeated engagement with firearms through collecting, handling, discussing, or researching them.  Langman (2009): He conceptualizes interest in firearms as a behavioral and motivational pattern reflected in acquiring weapons, studying past shootings, practicing with guns, or integrating firearms into personal fantasies or identity construction.  Leary et al. (2003): Leary includes interest in firearms when case materials document that the shooter kept, collected, admired, or frequently used weapons, or demonstrated a strong fascination with guns in conversations, journals, or behavior. |
| Cruelty to animals | Leary et al. (2003): Leary operationalizes cruelty to animals as documented engagement in intentional harm, injury, or mistreatment of animals, reported in investigative accounts, journalistic sources, or case documentation. |
| Deficits in coping skills | Lenhardt et al. (2018): Lenhardt conceptualizes deficits in coping skills as documented difficulties in managing stress, regulating emotions, and responding adaptively to interpersonal or academic challenges, as identified through case documentation and threat assessment materials. |
| Low resilience | Lenhardt et al. (2018): Lenhardt operationalizes low resilience as a diminished capacity to recover from stress, setbacks, or emotional disruption, identified through documented patterns in the shooters’ developmental, academic, and interpersonal histories. |
| TRAUMATIC EXPERIENCES AND HEALTH |  |
| Violent or abusive domestic environment | Dagenhard et al. (2019): Dagenhard operationalizes a violent or abusive domestic environment as documented exposure to physical, emotional, or psychological abuse within the home, identified through investigative materials, interviews, or official reports.  Farr (2019): Farr conceptualizes abusive home environments through a gendered lens, highlighting how chronic exposure to familial violence, intimidation, or emotional coercion contributes to a fractured sense of masculinity and identity.  Farr (2018): Domestic violence or abuse appears primarily in qualitative case descriptions when shooters experienced physical abuse, emotional neglect, or hostile household dynamics.  Freilich et al. (2022): Violent or abusive domestic environments are coded only when open-source accounts explicitly document domestic violence, child abuse, or neglect, either as direct victimization or as exposure to inter-parental violence.  Langman (2009): He defines this construct as ongoing exposure to severe physical, emotional, or sexual abuse, parental violence, substance-abusing caregivers, or chaotic home conditions marked by instability and aggression.  Paradice (2017): Paradice includes violent or abusive domestic environments when historical accounts identify childhood maltreatment, harsh punishment, exposure to domestic violence, or parental criminality. |
| At least one Adverse Childhood Experience (ACE) | Dowdell et al. (2022): Dowdell operationalizes ACE as the presence of at least one verified traumatic or harmful childhood experience occurring before age 18, identified through a systematic coding of media-reported life histories for each shooter. The ACE categories include physical, sexual, or psychological abuse; neglect; parental mental illness; problematic substance use within the household; parental separation or divorce; exposure to domestic violence; incarceration of a household member; or placement in foster care. |
| Physical health diagnosis | Dowdell et al. (2022): The variable is coded as present when journalistic sources state that the individual had been diagnosed with a non-psychiatric medical condition prior to the attack, such as asthma, diabetes, seizure disorders, chronic pain conditions, or other significant health issues. |
